# HIV‐1 establishes immediate latency in T cells expressing the viral Nef protein

**DOI:** 10.1002/2211-5463.70186

**Published:** 2025-12-20

**Authors:** Cindy Lam, Ivan Sadowski

**Affiliations:** ^1^ Department of Biochemistry and Molecular Biology Molecular Epigenetics Group, Life Sciences Institute, University of British Columbia Vancouver Canada

**Keywords:** HIV‐1, Latency, Nef, RGH, T cells

## Abstract

A variety of dual reporter HIV‐1 derivatives have been developed that enable detection of infected cells independently from transcription initiated from the 5′ LTR promoter. These reporters enable isolation of cells that are latently infected with HIV‐1 provirus. We have previously described several dual reporter derivatives, including Red Green HIV‐1 (RGH), which expresses mCherry from a constitutive internal promoter and GFP from the 5′ LTR. A limitation of most dual HIV‐1 reporter derivatives, including RGH, is that the Nef ORF is often disrupted to accommodate insertion of the internal reporter. Consequently, the potential role of Nef for establishment of latency using these reporters has not been clarified. To address this issue, we created three different RGH derivatives (RGHI), which express Nef from IRES elements downstream of the mCherry internal promoter. We found that each of these derivatives produced Nef protein at higher levels than wild‐type LAI virus and that Nef expressed from each of the IRES elements was localized to the cell membrane. All of the Nef‐expressing RGHI reporter viruses formed latent and productive infections and could be reactivated from latency at similar levels as the parental RGH derivative. These results indicate that expression of Nef does not affect the capability of HIV‐1 to form latent infections. Furthermore, we propose that the RGHI derivatives described here represent novel tools to examine the role of Nef for HIV‐1 replication.

## Graphical abstract

Nef is a viral protein often omitted from HIV‐1 reporter viruses. Consequently, its role in viral latency is unclear. We developed three novel dual reporter HIV‐1 derivatives that express Nef and allow for detection of latent and productive infection. Using these reporters, we show that Nef does not affect the establishment of immediate viral latency.

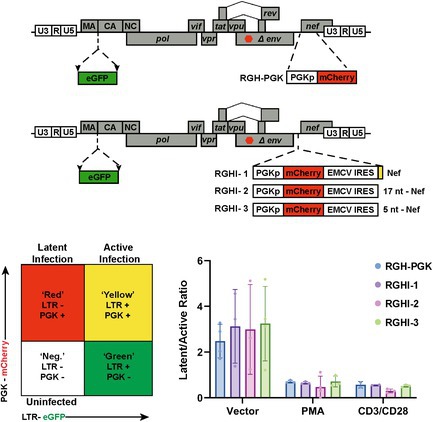

**Abbreviations**
ANOVA: analysis of variance
ART: antiretroviral therapy
ATCC: american type culture collection
BSA: bovine serum albumin
DAPI: 4′,6‐diamidino‐2‐phenylindole
DMEM: Dulbecco's modified Eagle's medium
DNA: deoxyribonucleic acid
DTT: dithiothreitol
EMCV: encephalomyocarditis virus
FBS: fetal bovine serum
FSC: forward scatter
GFP: green fluorescent protein
GTFs: general transcription factors
HIV‐1: human immunodeficiency virus type 1
HLA: human leukocyte antigen
HRP: horse radish peroxidase
IRES: internal ribosome entry sequence
LAI: lymphadenopathy‐associated virus
LTR: long terminal repeat
MOI: multiplicity of infection
ORF: open reading frame
PBS: phosphate buffered saline
PEI: polyethylenimine
PGK: phosphoglycerate kinase
PMA: Phorbol 12‐myristate 13‐acetate
RGH: red green HIV‐1
RGHI: red green HIV‐1 IRES
RIPA: radioimmunoprecipitation assay buffer
RNA: ribonucleic acid
RPMI: roswell park memorial institute
SDS/PAGE: sodium dodecyl sulfate/polyacrylamide gel electrophoresis
SSC: side scatter
TAR: tat‐responsive
TBS: tris‐buffered saline
TBST: tris‐buffered saline Tween 20
VSV‐G: vesicular stomatitis virus G

Despite significant advancements in antiretroviral therapy (ART), and three decades of intense research on HIV, there is currently not a cure for people infected with this virus. Upon infection, HIV‐1 integrates into the genome of target cells, predominately in introns of actively transcribed genes, where the newly integrated provirus becomes subject to regulation by cellular transcription factors [1]. The integrated provirus inevitably becomes transcriptionally repressed in unstimulated T cells, which produces a population of latently infected cells that acts as a reservoir for rebound of viral replication when ART is removed [2, 3]. Latently infected cells are not detected by the immune system, and the integrated provirus is unaffected by ART; this represents the major barrier toward a cure for infection [4, 5]. Consequently, a variety of strategies designed to eliminate the latently infected population have been proposed, many of which involve therapeutic modulation of latent provirus expression [1, 5, 6, 7, 8].

Development of these various potential therapeutic strategies has benefited from the use of cell line models of HIV‐1 latency where viral expression from stably integrated provirus is detected by fluorescent or luciferase reporter gene expression [9, 10, 11]. Furthermore, a variety of HIV‐1 reporter viruses have been developed that enable detection of infected cells independently from transcription initiated by the viral 5′ LTR [12, 13, 14]. These reporter viruses typically have an internal constitutive promoter, which expresses a fluorescent reporter protein or cell surface marker to mark infected cells, in addition to a reporter protein expressed from the HIV‐1 5′ LTR to indicate viral replication [12, 13, 14, 15]. The dual reporter viruses have contributed significantly to characterizing mechanisms regulating HIV‐1 latency because they enable characterization of factors associated with the LTR promoter in newly infected cells where provirus latency has been established [16, 17, 18, 19].

HIV‐1 inevitably forms latent provirus in CD4+ T cells that revert to quiescence; however, there appears to be several modes by which this occurs [20, 21]. In productively infected cells where newly integrated provirus becomes immediately transcriptionally active, viral expression is gradually shut down over the following days to weeks by epigenetic silencing mechanisms [22]. However, a surprising observation produced with dual reporter HIV‐1 derivatives was that the majority of newly infected cells establish transcriptionally silenced latent provirus within 24 h post infection [13, 15]. This effect was designated immediate or early latency [12, 15, 23] and was also shown to occur in normal CD4+ T cells with unmodified HIV‐1 [24]. The mechanism(s) contributing to immediate latency have not been determined, but the effect does not appear to be influenced by the site of chromosomal integration and is inhibited by elevated T‐cell signaling [23].

The first generation of dual reporter HIV‐1 derivatives typically have a frame shift mutation within *env* to prevent multiple rounds of infection but otherwise express all viral proteins apart from Nef [13, 15]. Nef function is not required for most aspects of HIV‐1 replication in T‐cell lines but is essential for viral pathogenesis *in vivo* [25, 26, 27]. Nef is a membrane‐associated myristylated protein, which serves as an adapter that interacts with various host proteins to interfere with multiple functions in infected cells [28]. Viruses with functional Nef are 10 times more infectious than those lacking Nef, largely because the protein down regulates host restriction factors [26, 29]. Nef defective viruses also cause delayed disease progression, highlighting its importance for pathogenicity [25]. It has also been shown that HIV‐1 Nef has a modest effect on latency establishment [30] and that Nef‐mediated HLA downregulation is correlated with viral reservoir size [31]. The Nef open reading frame is located at the 3′ end of the genome, partially overlapping the 3′ LTR of the integrated provirus, and is expressed early in infection from a subgenomic RNA spliced species [28]. Because of its position in the genome, and that the protein is considered dispensable for many aspects of HIV‐1 replication in cell lines, the Nef ORF was replaced with expression cassettes in many of the first generation dual reporter HIV‐1 derivatives [13, 14, 32]. Consequently, the effect that Nef function might have on establishment of latency using the dual reporters has not been clarified. To address this limitation, we produced modifications of our Red Green HIV‐1 dual reporters, which express all viral accessory proteins including Nef (RGHI). We found that the RGHI reporter viruses produce a similar proportion of productive and latent infections in Jurkat T cells, as does RGH following infection. Furthermore, HIV‐1 Nef+ reporter virus can be reactivated to a similar extent as Nef‐ virus upon T‐cell activation. These observations resolve an outstanding issue of whether Nef expression may alter the proportion of infected cells that establish immediate latent and productive infections shortly after infection. Furthermore, we suggest that the RGHI dual reporter derivatives may provide a novel alternative toward examining the function of Nef protein in latently infected cells.

## Materials and methods

### Generation of RGH Nef+ reporter virus derivatives

**Fig. 1:**
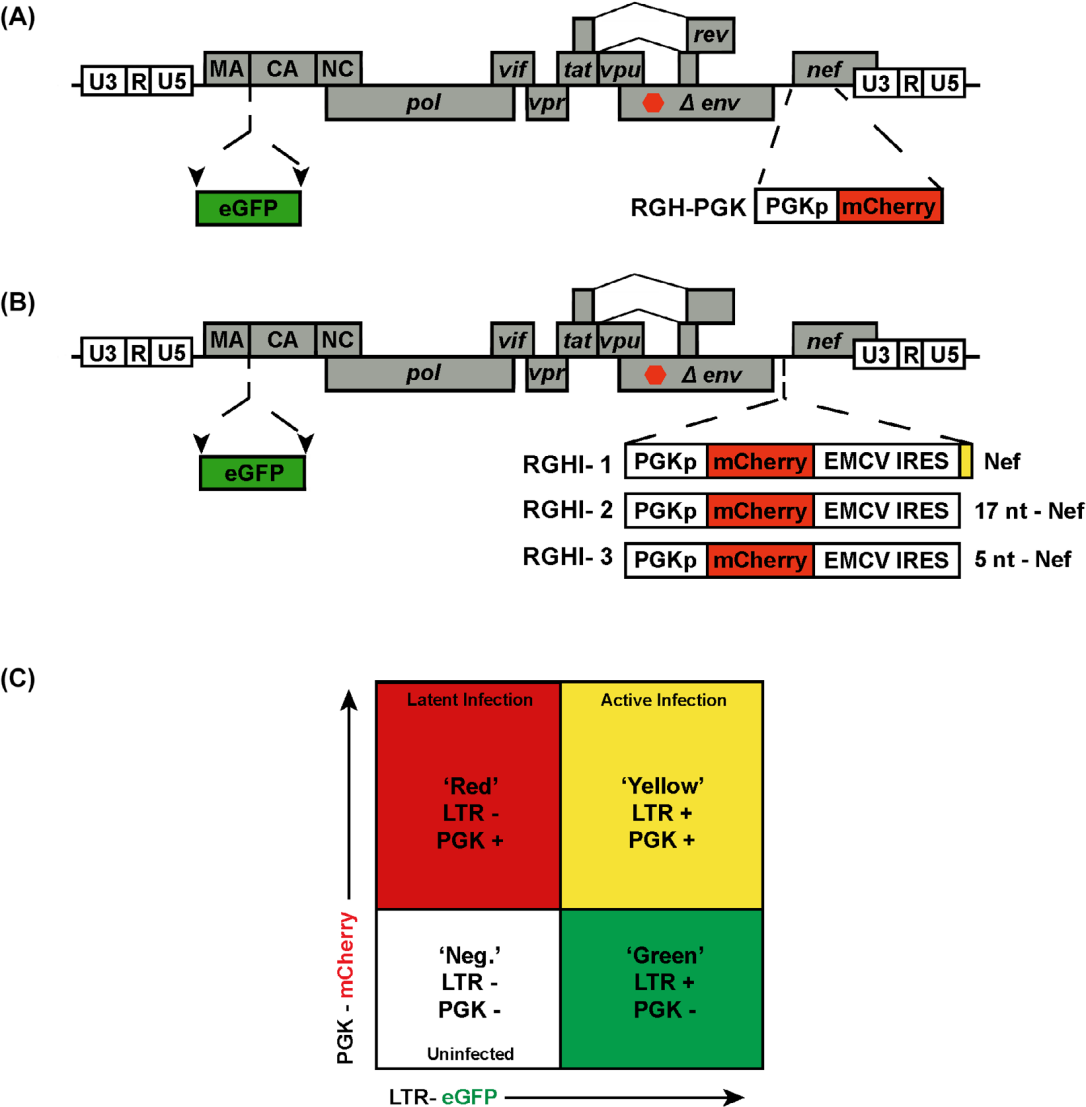
Schematic representation of the RGH3I dual reporter derivatives. Panel A, the RGH dual HIV‐1 reporter derivative expresses eGFP as fusion with gag peptides, flanked by HIV‐1 protease cleavage sites (arrows). Panel B, IRES elements were inserted immediately downstream of the PGK promoter–mCherry reporter between the *env* and *nef* open reading frames. RGH3I‐1 has a noncoding insert between an EMCV IRES lacking the endogenous translation start site and the Nef ORF. RGH3I‐2 and 3 have 17 and 5 nucleotides, respectively, between the IRES endogenous AUG and the Nef translational start site. Panel C, schematic depiction of FACS gating for RGH‐infected cell populations. Latently infected cells express mCherry but not eGFP (red), while productively infected cells express both mCherry and eGFP (yellow).

We produced three different versions of RGH with the IRES element from Encephalomyocarditis virus (EMCV) expressing Nef (Fig. 1B), expecting that at least one would produce functional Nef protein with an intact N terminus. RGHI‐1 has an eight nucleotide noncoding insert between the IRES and Nef ORF, where the minimal IRES translational start site was eliminated (Fig. S1). RGHI‐2 and RGHI‐3 position the Nef translational start site 17 and 5 nucleotides downstream of, and out of frame with, the minimal IRES translational start site, respectively (Fig. 1A, Fig. S1). The RGHI derivatives were constructed using the backbone plasmid pIS508, which is the original RGH vector where the 3′ region of the genome was replaced with pLAI *env*‐*nef*‐3′ LTR sequence, bearing a frame shift mutation in *env*, and which has a NotI/PacI/AscI linker inserted between the *env* and *nef* open reading frames using mutagenesis with the oligos IS2074 (CGTTAATTAACGGGCGCGCCTAAGATGGGTGGCAAGTGGTCAAAAAGTAG) and IS2065 (TTAACGGCGGCCGCTTATAGCAAAATCCTTTCCAAGCCCTGTCTTATTC). The PGK‐mCherry reporter was amplified from RGH‐PGK [32] and cloned into the NotI/PacI sites, and the EMCV IRES elements amplified from pHAGE NFKB‐TA‐LUC‐UBC‐dTomato‐W (49335; Addgene, Watertown, MA, USA) and cloned into the PacI/AscI sites. Complete sequences of all viral derivatives are available upon request.

### Cell culture, virus production and lentiviral transduction

Jurkat E6‐1 (RRID: CVCL_0367), CEM/C1 (RRID: CVCL_3496), and MOLT4 (RRID: CVCL_0013) cells were cultured in Roswell Park Memorial institute (RPMI) 1640 medium supplemented with 10% fetal bovine serum (FBS), penicillin (100 units·mL^−1^), streptomycin (100 g·mL^−1^), and L‐glutamine (2 mm). HEK293T (RRID: CVCL_0063) cells were cultured in Dulbecco's modified Eagle's medium (DMEM) supplemented with 10% FBS, penicillin (100 units·mL^−1^), streptomycin (100 g·mL^−1^), and L‐glutamine (2 mm). All cell cultures were obtained from ATCC, maintained in a humidified 37 °C incubator with a 5% CO_2_ atmosphere, and routinely tested for mycoplasma. Cell line authentication was routinely performed using morphology check by microscope. Vesicular stomatitis virus G (VSV‐G) pseudotyped viral stocks were produced by transfecting HEK293T cells with a combination of viral molecular clone and pHEF‐VSVg at a 9 : 1 ratio. Transfections were performed with polyethylenimine (PEI) at a ratio of 3 : 1 (PEI: DNA) in Gibco Opti‐MEM. For infection, 5 × 10^5^ cells were subjected to spinoculation for 1.5 h at 1500 rpm in 12‐well plates in 1 mL media containing 4 μg·mL^−1^ polybrene and viral supernatant ranging from 5 to 100 μL and flow cytometry was used 4 days post infection to determine viral titer. Cells were then infected in the same manner for an average multiplicity of infection (M.O.I) of 0.1. For cell stimulation, infected cells were treated with 12‐myristate 13‐acetate (PMA) or activated using the Human Anti‐CD3/CD28 T Cell Activation Kit (#70976; Cell Signaling Technology, Boston, MA, USA) for 18 h where indicated.

### Immunoblotting, immunofluorescence and flow cytometry

HEK293T cells were transfected with 2 μg of each plasmid, then 2 days post transfection, were lysed in RIPA buffer supplemented with 1× protease inhibitor cocktail and 1 mm DTT. Lysates were mixed with 4× SDS/PAGE sample buffer and boiled for 3 min. Protein concentrations were determined via Bradford assay, and 30 μg of total protein per sample was resolved on 15% SDS/PAGE. Samples were then transferred to 0.2 μm nitrocellulose membranes for 1 h 30 min at 100 V constant voltage. The membranes were blocked with 3% non‐fat milk (w/v) in TBS supplemented with 0.01% Tween‐20 (TBST), then incubated with primary antibody overnight at 4 °C in 3% milk in TBST. Membranes were washed with TBST and incubated with secondary antibody for 1 h at room temperature prior to imaging. Antibodies used were as follows: Tubulin (1 : 150 000; Abcam #ab6160, Waltham, MA, USA), Nef (1 : 1000; Abcam #ab63918), Goat Anti‐Rabbit HRP (1 : 2 000 000; Abcam #ab6721), Goat Anti‐Rat HRP (1 : 150 000; Abcam ab97057).

For analysis of fluorescent protein expression in infected cells, Jurkat cells were collected 4 days post infection and suspended in PBS. GFP and mCherry expression were measured using a Guava easyCyte instrument, and thresholds of forward scatter (FSC) and side scatter (SSC) were set to count live cells only. Data were analyzed using FlowJo (TreeStar). For immunofluorescence of Nef localization, HEK293T cells were grown on coverslips prewashed with 70% ethanol and coated with gelatin in tissue culture‐treated six‐well plates. Cells at 60–70% confluence were transfected with 2 μg DNA for RGH3I 1–3 vectors, or 3 μg for the wild‐type LAI vector, using PEI at a ratio of 3 : 1 (PEI: DNA). Two days post transfection, cells were fixed with 4% paraformaldehyde for 15 min. After fixation, cells were washed in PBS, and the plasma membrane labeled with wheat germ agglutinin‐Alexa Fluor 488 conjugate (5 μg·mL^−1^; ThermoFisher W11261, Waltham, MA, USA). Cells were washed, then permeabilized with 2.5% Triton X‐100 for 3 min, followed by two more washes with PBS. Samples were blocked with 5% BSA in PBS for 20 min before incubating the coverslips with mouse anti‐Nef antibody (1 : 200; NIH HIV Reagent Program ARP‐3689) in 5% BSA for 1 h. The samples were washed twice before incubation with donkey anti‐mouse conjugated Alexa Fluor 647 secondary antibody (1 : 1000; ThermoFisher #A31571) for 1 h. Finally, the samples were counterstained with 300 nm DAPI for 5 min and washed twice before assembling slides with 90% glycerol. Fluorescent images were collected using a Leica SP5 inverted laser scanning confocal microscope and analyzed using ImageJ.

### Statistics and reproducibility

Replicates are technical replicates and presented as mean values with ± standard deviation shown by error bars. *P*‐values were determined by performing two‐way ANOVA, as indicated, with the use of GraphPad Prism 10.0. Statistical significance is indicated as * *P* < 0.05, ** *P* < 0.01, or ns *P* > 0.05.

## Results

### Production of RGH derivatives expressing Nef

We constructed derivatives of our RGH‐PGK (Fig. 1A) dual reporter HIV‐1 virus that could express the Nef accessory factor. For this purpose, we inserted internal ribosomal entry sequence (IRES) elements immediately downstream of the mCherry internal reporter, which is constitutively expressed from the PGK promoter (Fig. 1B). Because Nef requires N‐terminal myristylation for function, the N‐terminal coding sequence cannot be perturbed. Consequently, we constructed three versions which slightly alter the Nef start codon relative to the EMCV IRES (Fig. 1B, RGHI‐1, 2 and 3), which can produce variable results with respect to translation start site and levels of expression of the downstream open reading frame [33, 34]. In RGH, expression from the 5′ HIV‐1 LTR is reported by eGFP, produced as a fusion with gag which is processed to form the matrix and capsid protein and release eGFP (Fig. 1A,B). Cells infected with RGH constitutively express mCherry from the internal phosphoglycerate kinase (PGK) promoter; consequently, newly infected cells that establish expression from the 5′ HIV‐1 LTR immediately (productive infection) express both GFP and mCherry (yellow, Fig. 1C), whereas cells that establish immediate latent infections express mCherry from the PGK promoter but not GFP (Red, Fig. 1C) [13, 23]. All of the RGHI derivatives also bear a frame shift mutation within *env* to prevent multiple rounds of infection (Fig. 1A,B).

### RGHI dual HIV‐1 reporters produce functional Nef protein

**Fig. 2:**
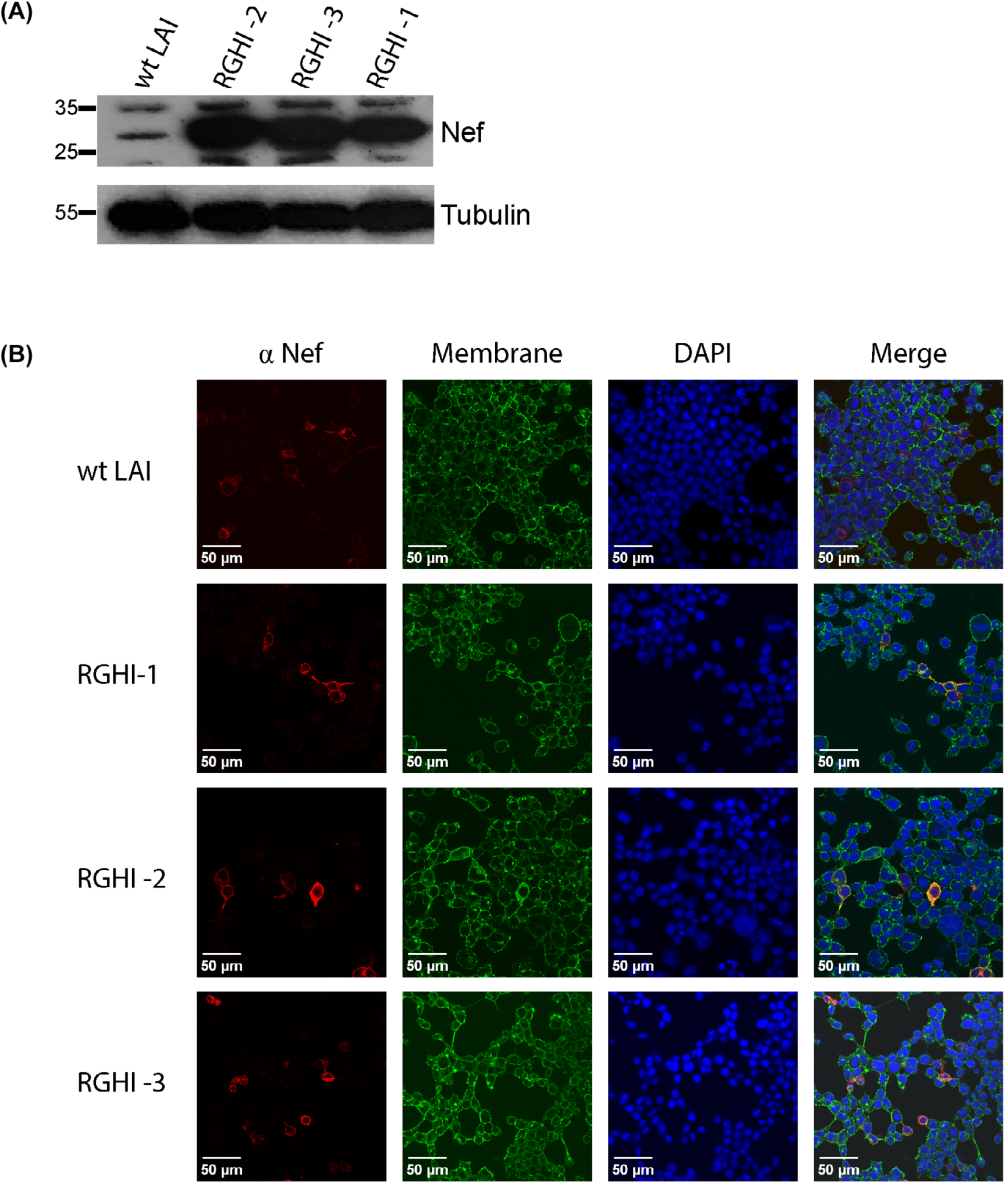
RGHI dual reporter derivatives express Nef protein. Panel A, expression of Nef in cells transfected with HIV‐1 reporter derivatives. HEK293T cells were transfected with 2 μg of wild‐type HIV‐1 LAI vector or the RGH3I 1–3 vectors. Cellular lysates were extracted 48 h post‐transfection and immunoblotted with an antibody against Nef or tubulin, *n* = 2. Panel B, Nef protein expressed from IRES elements is localized to the cell membrane. Transfected HEK293T cells were stained for Nef protein in permeabilized cells. Nuclei were counterstained with DAPI, and the plasma membrane was stained with a wheat germ agglutinin‐Alexa Fluor 488 conjugate, *n* = 3.

We first examined whether the RGH IRES derivatives could express Nef protein in transfections of HEK293T cells. We found that cells transfected with the RGH3I derivative constructs produced significantly greater amounts of Nef protein, relative to cells transfected with a plasmid (pLAI) bearing wild‐type HIV‐1 (Fig. 2A), indicating that all of the IRES derivatives express Nef protein at considerably higher levels than the unmodified HIV‐1 genome. Nef function requires N‐terminal myristylation, which targets the protein to the cell plasma membrane [28]. Consequently, we examined whether Nef protein expressed from the RGH IRES derivatives was localized to the cell membrane. For this, we examined Nef subcellular localization by immunofluorescence in HEK293T cells transfected with wild‐type HIV‐1 LAI or the RGH3I derivatives; for this experiment, the plasma membranes were labeled with a fluorescent wheat germ agglutinin, and the cells stained with anti‐Nef antibody and counterstained with a nuclear DAPI marker and examined using confocal fluorescent microscopy. In this analysis, we observed that Nef protein localized with the cell membrane for all RGH3I derivatives, similar to Nef expressed from wild‐type LAI (Fig. 2B). Much like the western blots in Fig. 2A, the intensity of the Nef signal was lower in the wild‐type LAI transfected cells compared to the RGHI derivatives; however, images were adjusted following acquisition to increase visibility for Nef produced by LAI. These observations indicate that Nef protein expressed from each of the IRES derivatives must have properly processed N termini.

### Expression of Nef does not affect establishment of HIV‐1 latency

**Fig. 3:**
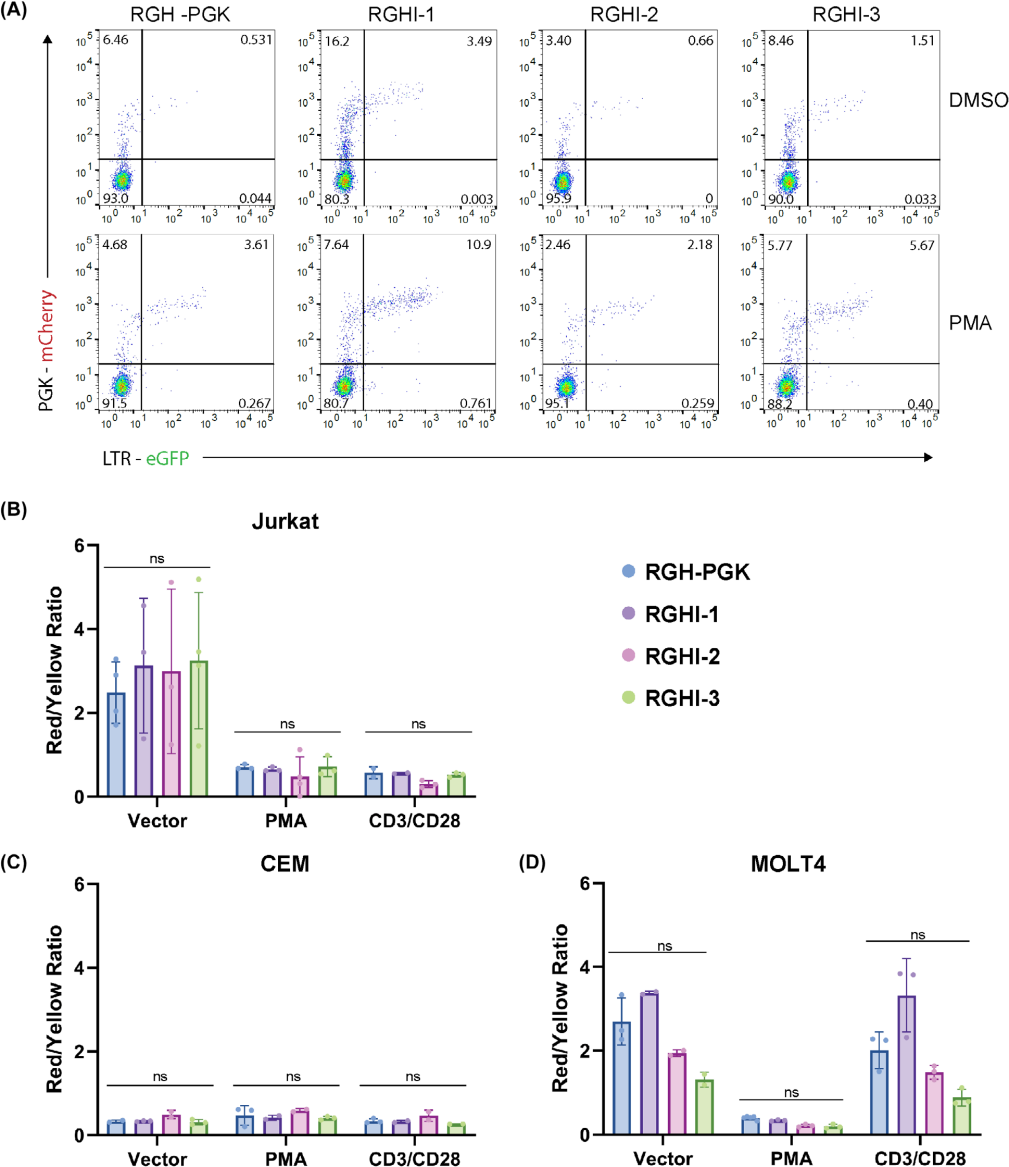
RGHI dual reporter derivatives expressing Nef establish immediate latency and are reactivated by T cell signaling. Panel A, Jurkat T cells were infected with RGH‐PGK and RGH3I 1–3. Three days post infection, the cells were treated with DMSO (top) or 10 nm PMA (bottom) for 18 h and were analyzed by flow cytometry for eGFP and mCherry expression. Panel B–D, Jurkat (B), CEM (C), or MOLT4 (D) cells were infected and treated as in Panel A. The proportion of latently infected cells, represented by the ratio of mCherry+ GFP‐ (Red)/mCherry+/GFP+ (Yellow), was determined 18 h following treatment with DMSO (left), PMA (middle) or CD3/CD28 cross‐linking (right). Error bars represent standard deviation, *n* = 3. ns, nonsignificant (two‐way ANOVA).

To determine whether expression of Nef affects the proportion of cells that establish immediate latency or productive infections, we performed flow cytometry on Jurkat cells infected with RGH or the RGHI dual reporter viruses. Cells were infected at low multiplicity of infection (M.O.I ~0.1) to ensure single infections per cell. Four days post infection, we found that the proportion of productive and latent infections were similar for all three RGH3I derivatives (Fig. 3A,B). Each of the RGHI dual reporter viruses produced a slightly greater proportion of immediate latent cells relative to RGH‐PGK, but this difference was not statistically significant (Fig. 3B). We also treated the cells with phorbol 12‐myristate 13‐ acetate (PMA), which stimulates protein kinase C and consequently several pathways that activate transcription from the HIV‐LTR [35]. In cells infected with all three RGHI derivatives we observed a similar decrease in the proportion of latently infected cells upon treatment with PMA, represented by a decrease in the ratio of cells expressing only mCherry relative to double positive mCherry/GFP cells. Again, each of the RGHI derivatives produced slightly lower proportions of latently infected cells upon PMA treatment compared to RGH, but this difference was not statistically significant (Fig. 3B). Similar effects on the proportion of latently infected cells were observed upon activation of the T‐cell receptor by CD3/CD28 crosslinking (Fig. 3B). We also characterized the RGHI dual reporters in CEM and MOLT4 T cells (Fig. 3C,D). The proportion of latently infected cells in CEM was consistent between RGH and the RGHI derivatives. However, we observed more productively infected cells compared to Jurkat cells. Consequently, activation via PMA treatment or CD3/CD28 crosslinking had little effect on the ratio of latent to productive infection in CEM cells (Fig. 3C). In MOLT4 cells, RGHI‐1 produced slightly higher proportions of latently infected cells compared to RGH, while RGHI‐2 and 3 resulted in lower proportions. Upon PMA treatment, RGHI‐2 and 3 also produced lower proportions of latently infected cells; however, these differences were not statistically significant, and because MOLT4 cells do not express surface CD3 [36], CD3/CD28 crosslinking had minimal effects on the ratios of latent and productive infection. Overall, these results indicate that expression of Nef by the dual reporter RGHI virus derivatives does not affect establishment of immediate latency or reactivation of latent HIV‐1 provirus.

## Discussion

A variety of dual reporter HIV‐1 derivatives have been developed to examine mechanisms regulating establishment of provirus latency [13, 15, 23, 37]. Most of these have disabled expression of the Nef protein, and consequently the role of Nef for establishment of latency with these reporter viruses has not been clarified. In this study, we demonstrate that three different dual reporter HIV‐1 derivatives that express Nef produce immediate latency at a proportion similar to the RGH‐PGK reporter, which does not express Nef protein. Furthermore, HIV‐1 expression in cells infected with the RGH derivatives expressing Nef was induced to similar levels as the virus that does not express Nef. These observations resolve an outstanding question as to whether establishment of early latency is affected by Nef function. This differs from previous research examining the role of Nef in viral latency that found HIV‐1 Nef causes a modest decrease in latency [30]. However, these studies examined reversion of actively replicating provirus to latency over the span of several weeks instead of immediate establishment of latency as we have examined here.

In this report we observed that the proportion of double negative (mCherry‐, GFP‐) was unaffected by PMA treatment of cells infected with the RGH‐PGK and the RGHI viruses. This differs from the original RGH virus, which has the cytomegalovirus (CMV) internal promoter instead of PGK [13], where we observed a decrease in the double negative population upon stimulation with PMA [23], which is likely caused by inactivation of the CMV promoter in these infected cells. This effect results in under representation of the infected population, which does not appear to be an issue with the RGH‐PGK derivatives described here.

Initial results with dual reporter HIV‐1 derivatives revealed that a significant proportion of newly infected cells establish latent provirus immediately upon infection [13, 14, 15], which is curious considering that the virus predominately integrates into introns of actively transcribed genes [38]. This suggests that the 5′ LTR on provirus that establishes immediate latency must be somehow precluded from recruiting the general transcription factors (GTFs) and RNA Polymerase II. Establishment of immediate latency does not appear to be affected by the site of chromosomal integration, but can be inhibited by elevated basal signaling downstream of the T cell receptor [23]. Immediate latent infections are also produced in cells constitutively expressing Tat protein [32], indicating that the block in transcription from the 5′ LTR likely occurs prior to synthesis of the nascent 5′ TAR RNA. One factor known to bind the 5′ LTR on latent provirus is the repressor YY1, which was identified as a repressor specifically bound in immediate latency [17], and consequently this factor may represent a defining switch that regulates establishment of latency of newly integrated provirus.

Several previous reports have described HIV‐1 reporter viruses that express Nef. The HIV‐rtTA derivative expresses a Tet‐On fusion, which enables doxycycline dependent replication of the virus. A modified form of this derivative was produced to express Nef from an EMCV IRES element, which enables more efficient replication in primary immune cells [39]. The CD8a‐d2EGFP‐ Nef+ reporter virus produces Nef from an IRES element downstream of a CD8a‐EGFP fusion that allows for purification of infected cells [40]. The HIV pMorpheus‐V5 dual reporter expresses Nef from an IRES element downstream from an HSA‐mCherry fusion, which is expressed from a natural subgenomic splice produced by 5′ LTR‐dependent transcription. Additionally, this reporter virus expresses V5‐NGFR from the PGK promoter, inserted into the env ORF, which enables detection of infection independently from 5′ LTR activity [37]. This reporter virus was also found to produce immediate latent cells in primary T cells, an effect that is associated with downregulation of CD4 expression [37]. Our results are consistent with these observations because we show that immediate HIV‐1 latency can be established in cells expressing Nef protein at higher levels than normally produced by wild‐type HIV‐1. We suggest that these novel RGHI derivatives will be useful for developing further insight into mechanisms regulating HIV‐1 latency, and the role of Nef protein in viral replication.

## Conflicts of interest

The authors declare no conflicts of interest.

## Author contributions

C. L. and I.S. conceived the experimental design. I.S. produced the RGHI reporter derivatives. C.L. performed all of the experiments. C.L. and I.S. wrote the manuscript.

## Supporting information


**Fig. S1.** Shown are junction DNA sequences for RGHI‐1, 2 and 3. The Nef ORF translational start site is indicated (boxed, green). The translational start site for the minimal [34] EMCV IRES is indicated (boxed, green).

## Data Availability

All data supporting the findings of this study are available in Figs 1, 2, 3 and the Supporting Information of this article. Full sequences of the RGHI derivatives are available from the corresponding author (sadowski@mail.ubc.ca) upon request.
