# Supplementary material for: HIV‐1 establishes immediate latency in T cells expressing the viral Nef protein
Source: FEBS Open Bio. 2025 Dec 20;16(6):1132–41. doi: 10.1002/2211-5463.70186 (PMC13238841; doi:10.1002/2211-5463.70186)
Supplement: Supplementary file 1 — Fig. S1. Shown are junction DNA sequences for RGHI‐1, 2 and 3. The Nef ORF translational start site is indicated (boxed, green). The translational start site for the minimal [34] EMCV IRES is indicated (boxed, green). [file FEB4-16-1132-s001.pdf]

Supplementary Figure 1

```
RGHI-1 GGGGACGTGGTTTTCCTTTGAAAAAGGCGCGCCTAA------------GATGGGTGGCAA
RGHI-2 GGGGACGTGGTTTTCCTTTGAAAAACACGATGATAATATGGCCACAACCATGGGTGGCAA
RGHI-3 GGGGACGTGGTTTTCCTTTGAAAAACACGATGATAC------------CATGGGTGGCAA
```

Minimal IRES TSS

Nef TSS
